# IL-17A expression in the adenoid tissue from children with sleep disordered breathing and its association with pneumococcal carriage

**DOI:** 10.1038/s41598-018-35169-x

**Published:** 2018-11-13

**Authors:** Chien-Chia Huang, Pei-Wen Wu, Chyi-Liang Chen, Chun-Hua Wang, Ta-Jen Lee, Chi-Neu Tsai, Cheng-Hsun Chiu

**Affiliations:** 1 Division of Rhinology, Department of Otolaryngology, Chang Gung Memorial Hospital and Chang Gung University, Taoyuan, Taiwan; 2 Graduate Institute of Clinical Medical Sciences, College of Medicine, Chang Gung University, Taoyuan, Taiwan; 3 Department of Otolaryngology–Head and Neck Surgery, Chang Gung Memorial Hospital and Chang Gung University, Keelung, Taiwan; 4 Molecular Infectious Disease Research Center, Chang Gung Memorial Hospital, Taoyuan, Taiwan; 5 Department of Thoracic Medicine, Chang Gung Memorial Hospital and Chang Gung Memorial Hospital and Chang Gung University, Taoyuan, Taiwan; 6 Division of Pediatric Infectious Diseases, Department of Pediatrics, Chang Gung Memorial Hospital and Chang Gung University, Taoyuan, Taiwan

## Abstract

Tonsil and adenoid-tissue hypertrophy (AH) is the most common cause of pediatric sleep-disordered breathing (SDB), with AH possibly initiated by repeated exposure to infectious agents or allergens. Here, we evaluated IL-17A activity in adenoid tissue from children with SDB and its association with AH and pneumococcal carriage. Thirty-five children (aged 3–12 years) with SDB and receiving adenoidectomy and tonsillectomy were enrolled. During surgery, nasopharyngeal carriage was determined by bacterial culture and multiplex PCR via nasopharyngeal swab, and adenoid samples were collected. IL-17A and associated cytokine expression was evaluated by real-time PCR and western blotting. The mRNA analysis showed that *IL-17A* level, *IL-17A*:*IL-10* ratio, and *RAR-related orphan receptor-γt:forkhead box P3* ratio were significantly higher in adenoid tissues with AH, as were *IL-17A* level and *IL-17A*:*IL-10* ratio in adenoid tissues with pneumococcal carriage. Additionally, pneumococcal carriage was more common in nasopharyngeal adenoids from patients without AH than those with AH. IL-17A was upregulated in adenoid tissues from patients with AH and with pneumococcal carriage. These results suggested that pneumococcal carriage initiates an IL-17A-mediated immune response in nasopharyngeal adenoids, which might be associated with AH in patients with SDB.

## Introduction

Obstructive sleep-disordered breathing (SDB) is a clinical condition associated with breathing problems due to an obstruction of the upper airways when sleeping and ranges in severity from simple snoring to obstructive sleep apnea syndrome (OSAS)^1,2^. SDB is common in the pediatric population, with an estimated prevalence of simple snoring in children at ~8% to ~27% and of OSAS at ~1% to ~5%^3,4^. Pediatric SDB can significant impact quality of life and link to behavioral problems, including hyperactivity, emotional irritability, and aggression, as well as failure to thrive if untreated^5–7^.

Hypertrophy of the tonsils and adenoid tissue (AH) is considered among the most common etiologies of SDB in children^2,8^ and causes narrowing of the upper airway, potentially leading to partial or complete obstruction of the airway particularly during sleeping as the pharyngeal muscle relaxes. As a result, tonsillectomy and adenoidectomy are considered the treatments of choice for most cases of pediatric SDB^8,9^. Moreover, regrowth of adenoid tissue is a common reason for residual or recurrent disease^10^. It is currently believed that AH is initiated by repeated exposure to infectious agents or allergens; however, the exact mechanism associated with the selective susceptibility of some children who have developed AH remains unknown.

Nasopharyngeal adenoids are a part of Waldeyer’s ring and represent a mass of lymphoid tissue located in a critical position in the upper respiratory tract and serve as the first line of immune defense and important effectors in both mucosal and systemic adaptive immunity^11^. In the lymphoid tissue of the adenoid, B lymphocytes represent ~65% of total lymphocytes, with the remaining comprising either T lymphocytes or plasma cells^12^. Among these, helper T (Th) cells play a critical role in immune protection based on their ability to activate B cells to produce antibodies and recruit various leukocytes to sites of infection and inflammation^13^. Th cells can differentiate into parallel types of effector CD4+ T cells (Th1, Th2, and Th17 subsets) to protect against different kinds of pathogens by stimulating the production of different cytokines and other soluble and cell-bound products and can function as immune effectors to eliminate infected cells^14^. Regulatory T (Treg) cells expressing the transcription factor forkhead box P3 (Foxp3) play an important regulatory role during infections, diminish immune responses to microbial pathogens, and prevent inflammation-related local-tissue damage or autoimmunity, but are also capable of contributing to infection chronicity^15,16^.

*Streptococcus pneumoniae* (pneumococcus) is among the most frequent colonizer of the pediatric nasopharynx and acts as a prerequisite of infectious diseases, including local mucosal diseases of the otitis media, sinusitis, and invasive diseases associated with pneumonia, bacteremia, and meningitis^17,18^. Recent evidence suggests an important role of Th17/Treg cells and their associated cytokines in the response to pneumococcal colonization in human nasopharyngeal adenoids^15,19^. Th17 cells expressing the transcription factor RAR-related orphan receptor-γt (RORγt) and its signature cytokine IL-17A play an important role in host defense against infection, including mucosal clearance of pneumococcal colonization^20–22^. However, Treg cells in the adenoid are also reportedly associated with persistent pneumococcal carriage in the nasopharynx^15,20^. Therefore, the relationship between Th17/Treg-mediated immune response, pneumococcal carriage, and AH remains incompletely understood. Here, we tested the hypothesis that Th17 cells and the associated immune response might play a role in *S. pneumoniae* scavenging and AH development by evaluating IL-17A expression in adenoid tissue from children with SDB and its association with AH and pneumococcal carriage.

## Methods

### Patients

Between April 2015 and December 2016, we prospectively recruited patients with SDB that were being managed at our respective Otolaryngology Departments (Chang Gung Memorial Hospital and Chang Gung University, Taoyuan, Taiwan). The inclusion criteria included the following pediatric patients: (1) aged 3 to 12 years; (2) exhibiting significant symptoms of snoring that disrupted the quality of sleep or daily activities, and (3) who planned to undergo adenoidectomy and tonsillectomy. Exclusion criteria included the following: (1) receipt of antibiotic therapy within the previous 4 weeks; (2) congenital anomalies to include cleft palate, Down syndrome, congenital heart disease, and craniofacial anomalies; or (3) major medical disorders, such as diabetes, nephrotic disease, autoimmune disorders, immunodeficiency, malignancy, and other chronic illnesses.

**Figure 1:**
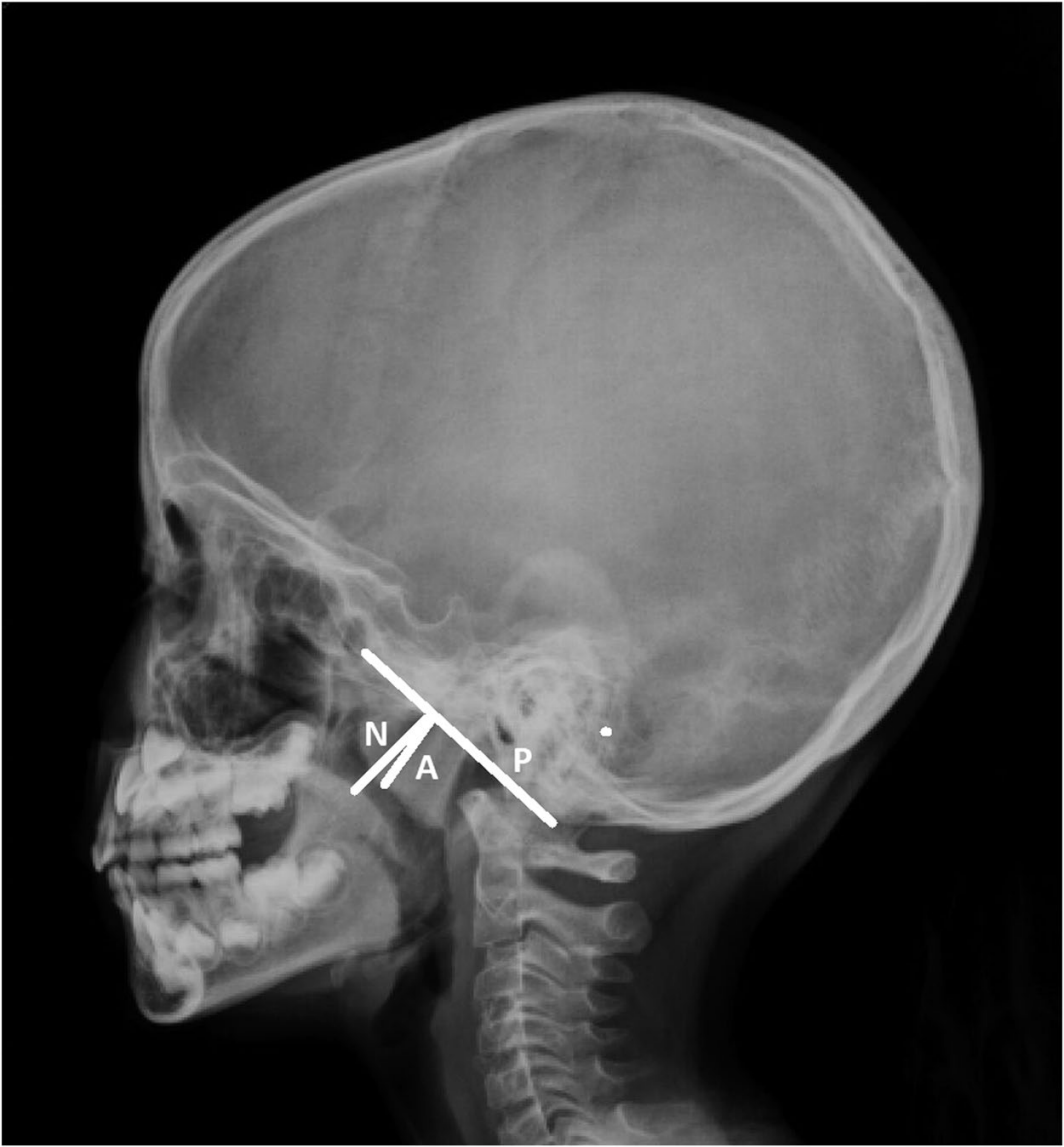
Adenoid: Nasopharynx ratio was measured by lateral cephalometry and was calculated as the ratio of the distance between the outermost point of anterior convexity of the adenoid shadow (A) and the straight part of the anterior margin of the basicocciput (P) to the distance between sphenobasioccipital synchondrosis and the posterior end of the hard palate (N).

SDB was diagnosed based on clinical symptoms, such as sleep disturbances, physical symptoms, emotional symptoms, daytime function, and polysomnography, if available. The size of nasopharyngeal adenoids according to skull lateral-view radiography, allergy profiles, symptom scores according to OSA-18 questionnaire^23^, and vaccination history for pneumococcal conjugate vaccine (PCV) were obtained before surgery. Adenoid:Nasopharynx (A:N) ratio was measured by lateral cephalometry, as described by Fujioka *et al*.^24^ and was calculated as the ratio of the distance between the outermost point of the anterior convexity of the adenoid shadow and the straight part of the anterior margin of the basicocciput to the distance between sphenobasioccipital synchondrosis and the posterior end of the hard palate (Fig. 1).

All patients were followed clinically for at least 6 months after surgery. All participants and/or their legal guardians provided informed consent prior to being enrolled in this study. The Institutional Review Board of Chang Gung Memorial Hospital approved the study (IRB number: 103–4773B). All research was performed in accordance with the relevant guidelines and regulations.

### Detection of nasopharyngeal colonization

Bacterial cultures were obtained using sterile swabs from the surface of the nasopharyngeal adenoid before removal of adenoid tissue during surgery. Swabs were transported to the laboratory in Amies transport medium (Copan Italia, Brescia, Italy) and placed on blood agar plates, eosin methylene blue plates, and Columbia colistin-nalidixic acid agar biplates and cultured at 37 °C for 48 h. *S. pneumoniae*, *Haemophilus influenzae*, *Moraxella cattarrhalis*, and *Staphylococcus aureus* isolates were identified by colony morphology and conventional methods of determination^25^.

Another nasopharyngeal swab was sent to the laboratory for *S. pneumoniae* serotyping, which was determined by multiplex PCR, as previous described^26^. Briefly, nucleic acids from each nasopharyngeal swab were extracted using a QIAamp genomic DNA kit (Qiagen, Valencia, CA, USA) according to manufacturer protocol, and the extracted DNA suspension was kept frozen at −70 °C until further use. Thirty five serotype-specific primer pairs were designed, as previously described^26^. A primer pair targeting *cpsA* found in all 90 known pneumococcal serotypes was used as the positive control. PCR conditions were 94 °C for 4 min, 30 cycles of 94 °C for 45 s, 54 °C for 45 s, and 65 °C for 2 min 30 s. PCR products were analyzed by gel electrophoresis on a 1.4% agarose gel at 120 V for 45 min, followed by staining with ethidium bromide and visualization by ultraviolet transillumination. All oligonucleotide primer sequences have been published by the Centers for Disease Control (Atlanta, GA, USA) and are available (http://www.cdc.gov/ncidod/biotech/strep/pcr.htm).

### Specimen collection and processing

Adenoid tissues were obtained by adenoidectomy. Adenoidal specimens were rinsed in phosphate-buffered saline (pH 7.6), stored at −70 °C, and then processed for real-time PCR and western blot.

### RNA extraction and reverse transcription

Total RNA was isolated from adenoid tissue using the RNeasy mini kit (Qiagen) according to manufacturer instructions. Extracted RNA was quantified using a NanoDrop machine (Thermo Scientific, Barrington, Ill, USA) and stained with ethidium bromide to determine RNA integrity. Reverse transcription was performed with random hexamer primers using the high-capacity cDNA reverse transcription kit (Applied Biosystems, Foster City, CA, USA).

### Real-time PCR to determine cytokine and transcription-factor expression

Real-time PCR was performed by TaqMan assay using primers specific for target genes [Supplemental Table S1] and *glyceraldehyde-3-phosphate dehydrogenase* (*GAPDH*) on an Applied Biosystems 7500 fast real-time PCR system (Applied Biosystems). The amplification conditions comprised an initial incubation at 95 °C for 10 min, followed by 45 cycles of 95 °C for 10 s, 60 °C for 20 s, and 72 °C for 10 s, with a final cooling period at 40 °C. Each sample was run in triplicate in separate tubes to permit quantification of gene expression. The mean threshold cycle (Ct) values were normalized to *GAPDH*, and relative mRNA levels of target genes were analyzed by the 2^−∆∆Ct^ method.

### Western blot analysis of IL-17A

Total protein was prepared using 1% IGEPAL lysis buffer (Sigma-Aldrich. St. Louis, MO, USA). Cellular proteins (10 μg) were fractionated by SDS-PAGE, electroblotted onto polyvinylidene difluoride membranes, and antibodies against IL-17A (1:500; Santa Cruz Biotechnology, Dallas, TX, USA) and β-actin (1:20,000; Millipore, Billerica, MA, USA) were used to assess differences in protein levels by enhanced chemiluminescence. Protein bands were visualized using a gel documentation system (Alpha Innotech, San Leandro, CA. USA). Relevant band intensities were quantified by densitometric analysis and normalized to β-actin.

### Statistical analysis

Data were presented as the mean ± standard deviation and statistically analyzed using GraphPad Prism 5 software (GraphPad Software, San Diego, CA, USA). Categorical data were compared using the Chi-squared test or Fisher’s exact test, as appropriate. Continuous variables were analyzed by the Mann–Whitney *U* test when comparing between two groups. Correlation was determined using the Spearman’s correlation coefficient. Statistical significance was set at p < 0.05. The power calculated from the difference between the primary outcomes in the study groups was 83.7%.

## Results

### Clinical characteristics of the study population

A total of 35 consecutive SDB patients who received adenoidectomy and tonsillectomy during the study period were enrolled. Twenty six were presented with AH (SDBwAH) defined by an A:N ratio >60%. The remaining nine patients were grouped as SDB without AH (SDBsAH; A:N ratio <50%). Table 1 summarizes the clinical characteristics of the participants. There were no statistical differences between the two groups, except for the A:N ratio.

**Table 1:** Clinical characteristics of study populations.

	SDBwAH	SDBsAH	p value^†^
Case number	26	9	
Age (year)	6.7 ± 2.1	7.4 ± 2.4	ns
Male: Female	20: 6	8: 1	ns
BMI	17.6 ± 4.9	17.5 ± 2.6	ns
Atopy, n (%)	13 (50.0)	4 (44.4)	ns
PCV 7, n (%)	5 (19.2)	0 (0)	ns
PCV 13, n (%)	14 (53.8)	7 (77.8)	ns
OSA-18	68.0 ± 18.3	66.1 ± 17.8	ns
WBC (1000/dL)	8.3 ± 2.1	8.9 ± 2.1	ns
Eosinophil (%)	4.6 ± 3.8	3.7 ± 3.5	ns
Total IgE	377.3 ± 716.7	242.8 ± 184.2	ns
A:N ratio (%)	80.5 ± 7.9	46.6 ± 1.9	<0.001**

Data was represented as the mean ± standard deviation.

^†^Categorical variables were compared using the Chi-squared test or Fisher’s exact test, as appropriate, and continuous variables were analyzed by the Mann–Whitney *U* test between the two groups.

^**^p < 0.01.

SDBwAH, sleep-disordered breathing with adenoid hypertrophy; SDBsAH, sleep-disordered breathing without adenoid hypertrophy; BMI, body mass index; PCV, pneumococcal conjugate vaccine; OSA-18, Obstructive Sleep Apnea-18 questionnaire; A:N ratio, adenoid:nasopharynx ratio; ns: not significant.

### Cytokine and transcription-factor mRNA expression in nasopharyngeal adenoids

**Figure 2:**
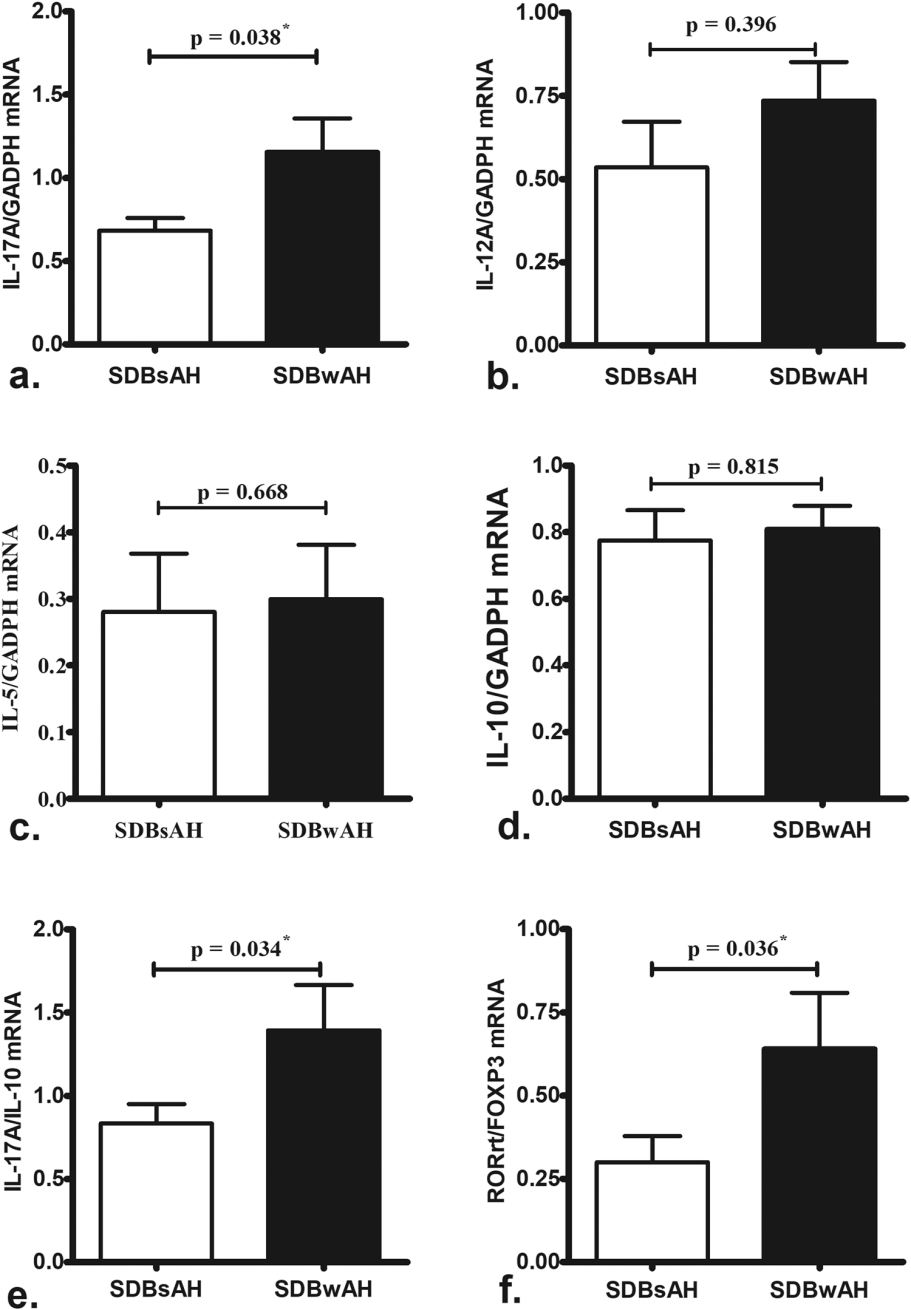
Comparison of *IL-17A* (**a**) *IL-12A* (**b**) *IL-5* (**c**) and *IL-10* (**d**) mRNA levels, as well as the *IL-17*:*IL-10* (**e**) and *RORγt*/*Foxp3* (**f**) ratios, in adenoid tissues between SDBwAH (*n* = 26) and SDBsAH (*n* = 9) patients according to real-time PCR. *IL-17A* mRNA levels and *IL-17*:*IL-10* and *RORγt*/*Foxp3* ratios were significantly upregulated in SDBwAH patients relative to SDBsAH patients. ^*^p < 0.05 according to Mann-Whitney *U* test.

The mRNA-expression levels of *IL-17A*, the *IL-17A*:*IL-10* ratio, and the *RORγt*/*Foxp3* ratio were significantly greater in adenoid tissues from SDBwAH patients as compared with those from SDBsAH patients (Fig. 2a,e,f). There was no differences in *IL-12A* (Th1-driven cytokine), *IL-5* (Th2-driven cytokine) and *IL-10* (Treg-driven cytokine) mRNA levels between SDBwAH and SDBsAH patients (Fig. 2b–d).

**Figure 3:**
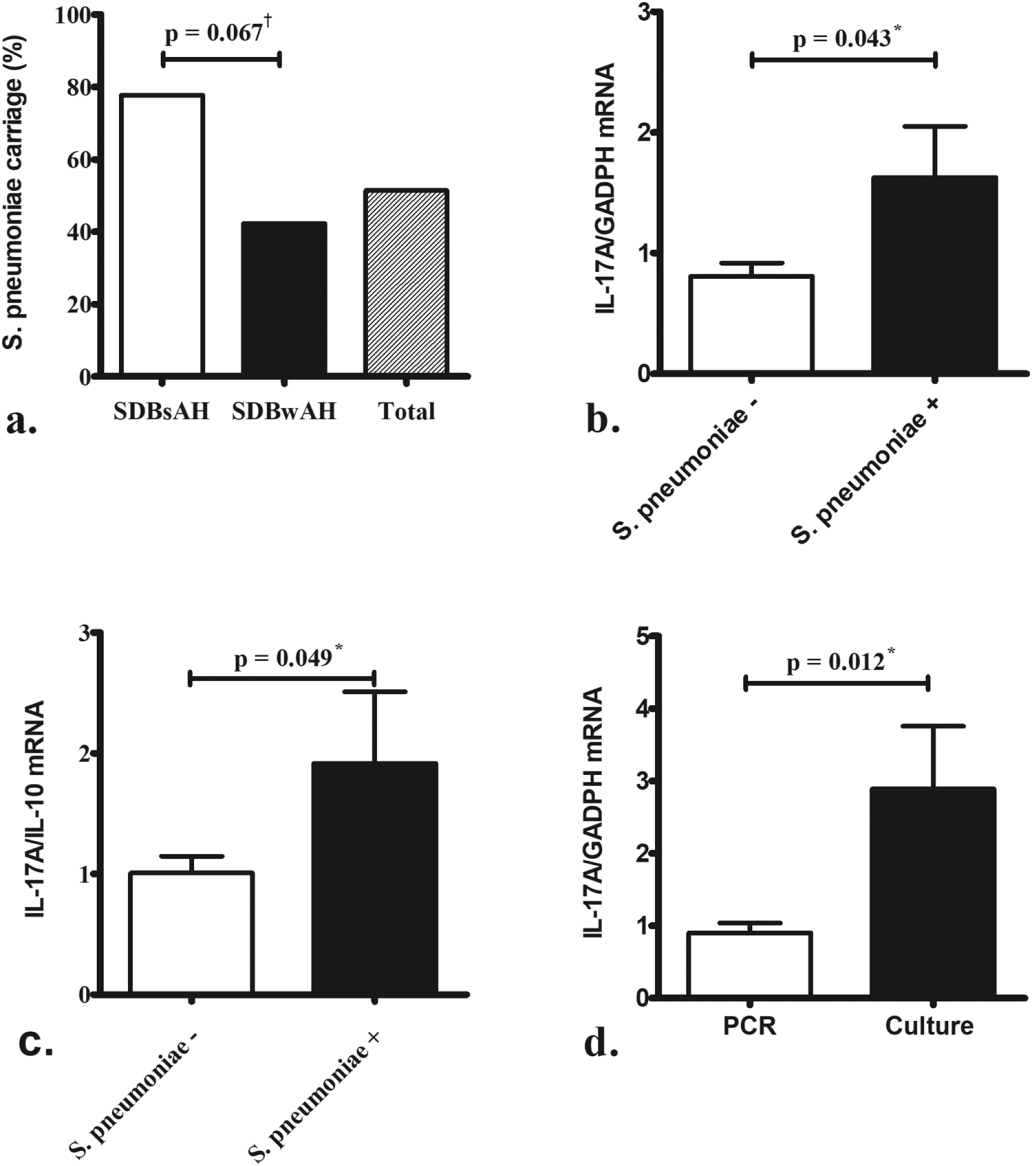
*Streptococcus pneumoniae* carriage was more common in nasopharyngeal adenoids of patients without AH (**a**). Significantly higher levels of *IL-17A* (**b**) mRNA levels and *IL-17*:*IL-10* (**c**) ratio were observed in adenoid tissue positive for pneumococcal carriage (*n* = 11) than that negative for pneumococcal carriage (*n* = 15) in patients with AH. Adenoid tissues harboring *S. pneumoniae* according to conventional culture (*n* = 4) expressed significantly higher levels of *IL-17A* mRNA as compared with those harboring *S. pneumoniae* detected by multiplex PCR (*n* = 7) (**d**). ^†^p < 0.1 according to the Chi-squred test; ^*^p < 0.05 according to the Mann-Whitney *U* test.

Pneumococcal carriage was more common in nasopharyngeal adenoids from patients without AH than in those with AH (Fig. 3a). Comparison of AH tissues positive and negative for pneumococcal carriage showed significantly higher levels of *IL-17A* (Fig. 3b) and IL-17A:IL-10 (Fig. 3c) mRNA in the 11 AH tissues positive for pneumococcal carriage than the other 15 AH tissues negative for pneumococcal carriage. Furthermore, adenoid tissues positive for *S. pneumoniae* as detected by conventional culture (either with or without positive PCR results) showed significantly higher levels of *IL-17A* mRNA than those only positive for *S. pneumoniae* according to multiplex PCR (Fig. 3d).

**Figure 4:**
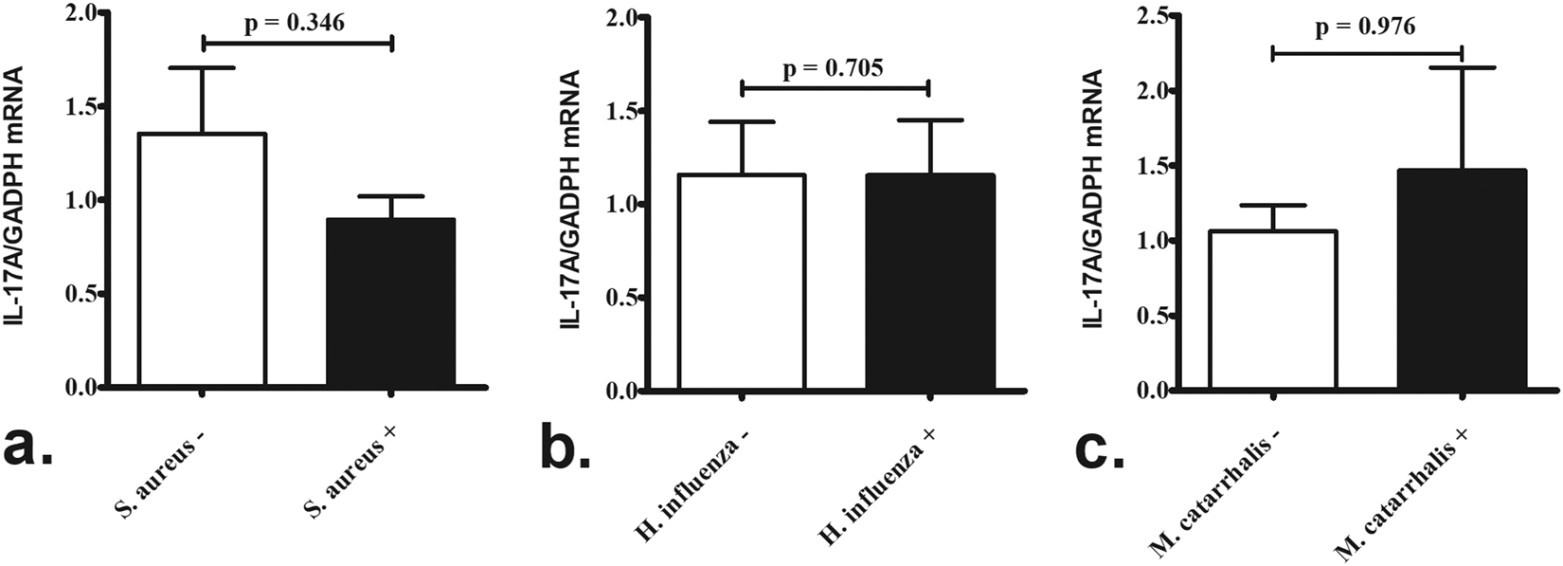
Comparison of *IL-17A* expression in adenoid tissue harboring different colonizers. There was no significant difference in *IL-17A* mRNA levels in adenoid tissue between children positive and negative for *Staphylococcus aureus* (*n = *14 and 12, respectively) (**a**) *Haemophilus influenzae* (*n* = 11 and 15, respectively) (**b**) or (**c**) *Moraxella cattarrhalis* (*n* = 6 and 20, respectively) colonization (determined from the results of conventional culture). P-values were analyzed by the Mann–Whitney *U* test.

Comparison mRNA expression in adenoid tissue harboring different colonizers showed no significant difference in *IL-17A* mRNA levels between children positive or negative for *S. aureus*, *H. influenzae*, or *M. catarrhalis* (Fig. 4) colonization (determined from the results of conventional culture).

### Western blot analysis of IL-17A

**Figure 5:**
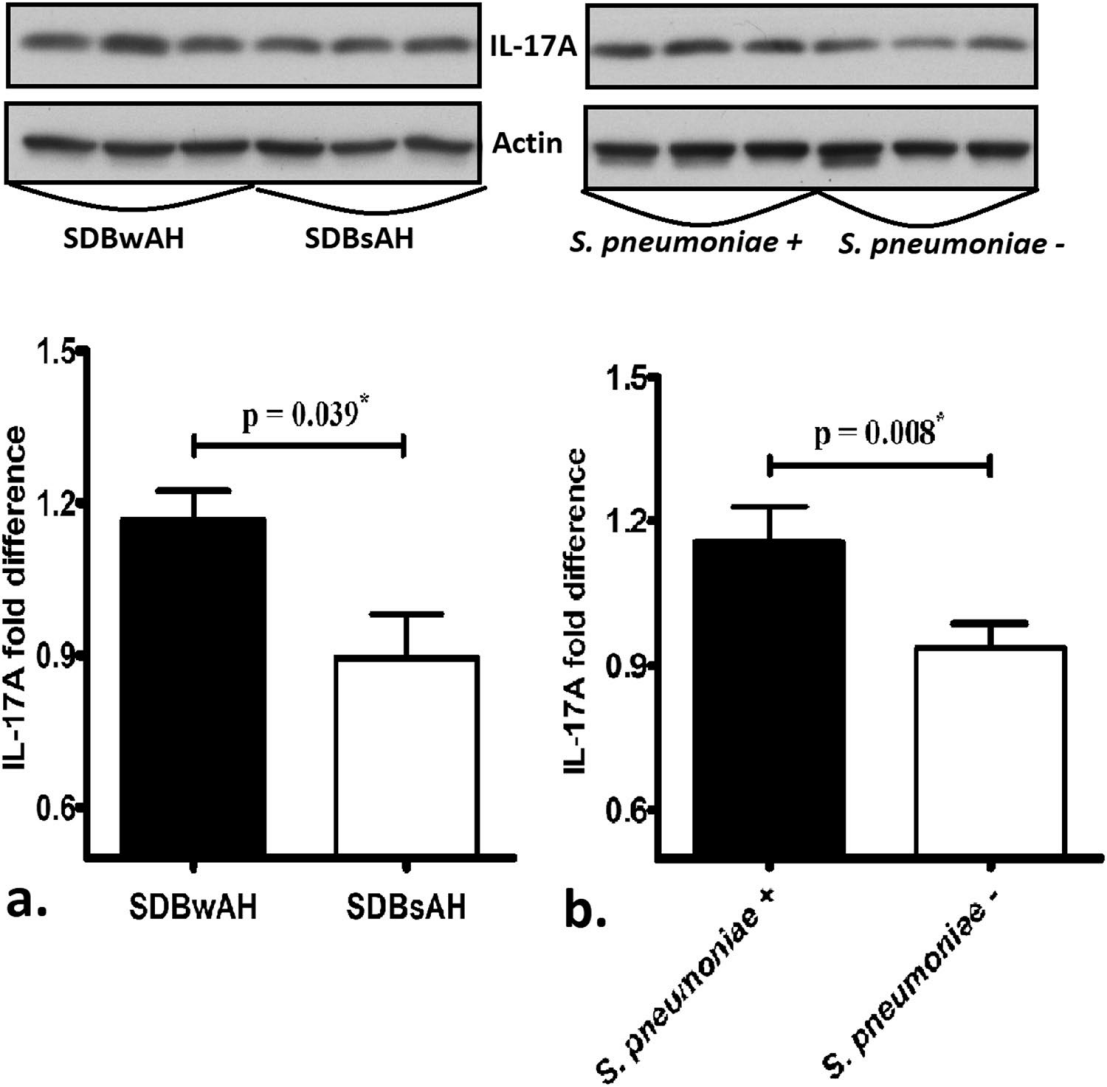
Protein quantification by western blot confirmed result of mRNA analysis, showing that adenoid tissue from SDBwAH patients and positive for pneumococcal carriage expressed higher levels of IL-17A than those from SDBsAH patients (**a**) and negative for pneumococcal carriage (**b**) respectively. The grouping of blots cropped from the same gel of the same patients. ^*^p < 0.05 according to the Mann–Whitney *U* test.

IL-17A levels in adenoid tissues from the 32 patients, measured by western blot, confirmed the results of mRNA analysis, showing that SDBwAH patients and those positive for pneumococcal carriage expressed higher levels of IL-17A relative to SDBsAH patients (Fig. 5a) and those negative for pneumococcal carriage (Fig. 5b) respectively.

### Association studies

**Figure 6:**
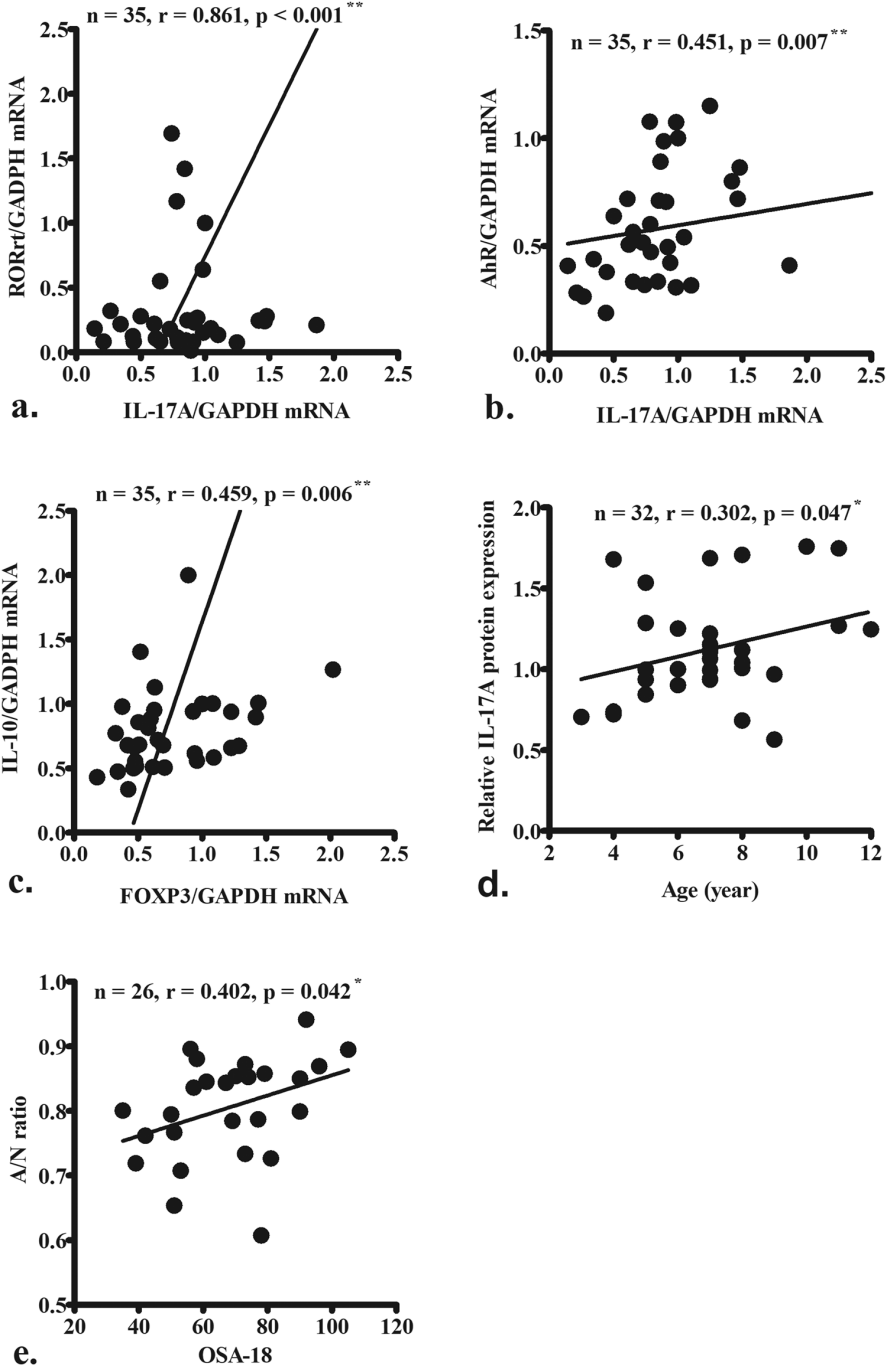
*IL-17A* mRNA expression was correlated with that of *RORγt* (**a**) and *AhR* (**b**). *IL-10* mRNA expression was correlated with that of *Foxp3* (**c**). *IL-17A* mRNA levels were positively correlated with the age of patients with SDB (**d**). The adenoid:nasopharynx (A:N) ratio was positively correlated with OSA-18 symptom scores in SDBwAH patients (**e**). Data were analyzed using Spearman’s correlation coefficient. ^*^p < 0.05; ^**^p < 0.01.

*IL-17A* mRNA levels were correlated with those of its transcription factors including *RORγt* and *aryl hydrocarbon receptor* (*AhR*) (Fig. 6a,b), whereas *IL-10* levels were correlated with *Foxp3* levels (Fig. 6c). Additionally, *IL-17A* levels were positively correlated with SDB patient age (Fig. 6d), which corresponded to our knowledge of that the pneumococcal carriage rate decreases with age. Furthermore, A:N ratio was positively correlated with OSA-18 symptom scores in SDBwAH patients (Fig. 6e), indicating the AH played a critical role in the severity of OSA.

## Discussion

This is the first study to evaluate IL-17A expression in adenoid tissue from children with SDB and its association with AH and pneumococcal carriage. The results revealed increased mRNA expression of *IL-17A*, *IL-17:IL-10* ratio, and *RORγt/Foxp3* ratio in adenoid tissues from SDBwAH patients and those positive for pneumococcal carriage as compared with that in SDBsAH patients and those negative for pneumococcal carriage. Additionally, *IL-17A* mRNA levels were correlated with those of its transcription factor *RORγt*, as well as that of *AhR*, and mRNA levels of *IL-10*, a cytokine associated with Treg cells, were correlated with those of its transcription factor *Foxp3*. Furthermore, pneumococcal carriage was more common in nasopharyngeal adenoids from SDBsAH patients as compared with those from SDBwAH patients. These results indicated that pneumococcal carriage might initiate IL-17A-mediated immune response in nasopharyngeal adenoids to eradicate colonization, and that this activity might be associated with AH in patients with SDB. Children with high levels of IL-l7A in response to pneumococcal carriage would likely exhibit an elevated ability to clear mucosal carriage; however, these children might also be susceptible to AH development due the activation of IL-17A-associated immune pathways.

Previous studies explored the role of Th17/Treg cells in pneumococcal carriage in the nasopharynx^15,19–22^. Pneumolysin, a protein toxin expressed by virtually all pneumococcal strains and considered an important virulence factor associated with invasive pneumococcal disease, reportedly activates Th17 cells in human nasopharynx-associated lymphoid tissue and that might be associated with mucosal clearance of pneumococcal carriage^22^. Jiang *et al*.^20^ reported that upregulation of Foxp3+ Treg cells might downregulate the production of Th17 cells in the adenoid, resulting in decreased scavenging of *S. pneumoniae* and chronic pneumococcal carriage. The balance between Th17 and Treg cells in the nasopharyngeal adenoid appears to be a major host factor closely associated with clearance of *S. pneumoniae* from the nasopharynx^27^. The present study directly investigated the expression of cytokines in adenoid tissue and revealed increased expression of *IL-17A*, as well an elevated *IL-17A*:*IL-10* ratio, in adenoid tissues positive for pneumococcal carriage. Furthermore, adenoid tissues positive for *S. pneumoniae* according to conventional culture (either with or without PCR-positive results) expressed significantly higher levels of *IL-17A* mRNA relative to those with *S. pneumoniae* detected merely by multiplex PCR. Although only four patients were positive for pneumococcal culture, the difference was significant (p = 0.012). PCR is generally considered more sensitive than conventional culture in detecting the presence of *S. pneumoniae*. This implicated a lower density of pneumococcal carriage detected by multiplex PCR than those by conventional culture. As a consequence, the IL-17A expression in culture-positive patients was higher. Our findings implicated that the bacterial density of pneumococcal carriage corresponded to *IL-17A* expression; however, Hoe *et al*.^28^ reported a different result, revealing that a high density of pneumococcal nasopharyngeal carriage was associated with reduced IL-17 secretion in blood samples and isolated peripheral blood mononuclear cells, whereas low pneumococcal-carriage density was associated with increased IL-17 secretion in children. This might be because IL-17A could be important for controlling the inflammatory process under conditions of low pneumococcal-carriage density^28^ However, dysregulated or ineffective IL-17A responses might also provide a favorable environment to allow S. *pneumoniae* to survive in high-density environments, thereby leading to frequent or chronic infectious diseases. It is possible that the types of specimens collected, the patients cohort, the disease setting, and patient socio-economic status might have contributed to the differences observed between the studies. In the present study, we directly measured the expression of cytokines and the presence of pneumococcal carriage in adenoid tissue obtained during surgery on SDB patients. We believe that these results better reflect actual local immune response in the nasopharynx and might correlate better with carriage status.

Although IL-17A was first described as the signature cytokine of Th17 cells^29^, group 3 innate lymphoid cells (ILC3s) have recently emerged as key producers of IL-17A^30,31^. ILCs are prevalent at mucosal sites and play an important role in the defense of mucosal surfaces^32^. Three ILC groups that parallel CD4+ Th lymphocyte subsets have been proposed^33,34^ and include ILC3s, which also express RORγt and produce IL-17A and IL-22 in response to mucosal infection^30^. Therefore, ILC3 might play a central role in the early regulation of antimicrobial activity and inflammation at mucosal sites, such as the intestine and respiratory tract^35^. Lymphoid-tissue-inducer (LTi) cells are principal members of ILC3s and usually aggregate with stromal, dendritic, and B cells in cryptopatches, isolated lymphoid follicles, or mature isolated lymphoid follicles and are associated with lymphoid-tissue development^36^. Consequently, our results suggest that activation of the IL-17A-mediated immune response by pneumococcal carriage might also by related to the stimulation of LTi cells, resulting in hyperplasia of adenoid tissue. However, further studies involving adenoid cell profiling or co-staining for ILC3s and Th17 cells are necessary to clarify the main IL-17A producers in these samples.

Recent evidence implicated inflammatory cytokines in the onset and progression of pediatric SDB or OSAS based on the concurrence of chronic systemic inflammation^37,38^. Previous studies reported increases in proinflammatory cytokines, including C-reactive protein and IL-17, in pediatric OSAS patients^39,40^, and Huang *et al*. showed that children with OSAS present with elevated serum IL-17 levels that might contribute to complicated prognosis of pediatric OSAS^41^. Additionally, Ye *et al*.^42^ demonstrated significant increases in peripheral Th17-cell number, Th17-related cytokines (IL-17 and IL-6), and *RORγt* mRNA levels in severe OSAS cases^42^. Most previous studies investigated the expression of these inflammatory cytokines or their mediators in peripheral blood^39–42^. Ni *et al*.’s study showed that Th17/Treg imbalance in pediatric peripheral blood and adenoid tissues was associated with increased risk of developing AH and OSA^43^. In pediatric patients, AH is the common cause of SDB or OSAS in children^2,8^. In this study, we also found that the A:N ratio was positively correlated with OSA-18 symptom scores in SDBwAH patients in our study cohort. Subsequent measurement of *IL-17A* in adenoid tissues suggested its important role in the development of AH and SDB, thereby supporting previous findings of IL-17A involvement in SDB and OSAS severity.

Longitudinal carriage studies of pneumococcus showed that pneumococcal colonization of the upper respiratory tract is a dynamic process, with most children colonized serially with single or even multiple serotypes^44^. Each colonization event might persist from days to months, thereby providing opportunities for infection or serving as a reservoir for the spread of the pathogen within the community. Pneumococcal carriage is common in young children and decreases with age. Mubarak *et al*.^27^ demonstrated a dynamic relationship between Th17 and Treg cells in the human nasopharynx that evolves with age, with increased Th17-cell and decreased Treg-cell number possibly corresponding to diminished pneumococcal carriage. Figure 6d shows increase with age was associated with increase in IL-17A expression in adenoids (with and without pneumococcal carriage). This result indicates that IL-17A activity increases with age, leading to a decline in the pneumococcal carriage rate. This support its role in protecting against pneumococcal colonization.

Th17 cells also play an important role in *S. aureus* infection. However, their role in healthy carriers remains relatively unstudied^45^. The Th1/Th17 profile was most common among T cell clones responding to extracellular *S. aureus* antigens. However, a recent study^46^ suggested specific differential host-strain interactions in *S. aureus*. Th17 counts and IL-17 levels in response to stimulation with endogenous strains (strains that were carried by individuals) were significantly lower than those in response to stimulation with exogenous ones (strains not carried by the individuals). In the present study, there was no difference in the mRNA levels of *IL-17A* (Fig. 4), the *IL-17A*:*IL-10* mRNA ratio, and the *RORγt*/*Foxp3* mRNA ratio (data not shown) between children positive or negative for *S. aureus* colonization. Taken together, these findings suggest that colonized *S. aureus* (endogenous strains) may have low or no impact on the expression of IL-17A in healthy subjects. Furthermore, the co-colonization of *S. aureus* was found in six of 11 children with *S. pneumoniae* colonization and in eight of 15 children without *S. pneumoniae* colonization in this cohort. There was no difference in the frequency of the co-colonization of *S. aureus* between the two groups (p = 0.951 by Chi-Square analysis). Based on these results, we could omit the confounding effect of *S. aureu*s.

SDB is common in the pediatric population and inked to impaired life quality, behavioral problems, and failure to thrive^5,6^. Nasopharyngeal carriage of *S. pneumoniae* is also prevalent in young children and associated with both local and invasive infection^17,18^. Therefore, defining the role of IL-17A and its associated immune pathway in AH and pneumococcal carriage is important to support the design, implementation, and tailoring of therapeutics for successfully treating and preventing these diseases. This study expands the understanding of the possible mechanisms associated with AH and pneumococcal carriage. Future novel therapeutics targeting the IL-17A-related inflammatory response or capable of modulating the function of Th17 cells might be beneficial for those with residual or recurrent SDB following adenoidectomy and tonsillectomy. Moreover, our findings will support the development of new strategies to prevent pneumococcal-related diseases, possibly through the use of a whole-cell pneumococcal vaccine^47^.

This study has several limitations that warrant consideration. First, the study was conducted in a relatively small number of children, and our results need to be confirmed in larger studies. Second, this study implicated an association between IL-17A-related immune response, pneumococcal carriage, and AH; further experimental study involving experimental stimulation or inhibition based on animal models will be necessary to clarify the causative relationships and associated mechanisms implied by our findings. Finally, long-term studies are needed to investigate the continuing impact of IL-17A activity on the clinical outcomes of patients with AH or pneumococcal carriage.

## Conclusion

Here, we showed that IL-17A was upregulated in adenoid tissues derived from SDBwAH patients positive for pneumococcal carriage. Our findings indicated that pneumococcal carriage was more common in the nasopharyngeal adenoids of SDBsAH patients as compared with those with SDBwAH. These result suggested that pneumococcal carriage initiates an IL-17A-mediated immune response to eradicate *S. pneumoniae* colonization in nasopharyngeal adenoids, which might be associated with AH in patients with SDB. Future novel therapeutics targeting IL-17A-related immune responses might be beneficial for treating these diseases.

## Electronic supplementary material

Supplementary information

## Data Availability

All data described in the study are presented in the manuscript. The datasets analyzed are available from the corresponding author on reasonable request.
