# Supplementary material for: IL-17A expression in the adenoid tissue from children with sleep disordered breathing and its association with pneumococcal carriage
Source: Sci Rep. 2018 Nov 13;8:16770. doi: 10.1038/s41598-018-35169-x (PMC6233154; doi:10.1038/s41598-018-35169-x)
Supplement: Supplementary file 1 — Supplementary information [file 41598_2018_35169_MOESM1_ESM.pdf]

IL-17A expression in the adenoid tissue from children with sleep disordered breathing and its association with pneumococcal carriage

Chien-Chia Huang[1,2]; Pei-Wen Wu[1,3]; Chyi-Liang, Chen[4]; Chun-Hua Wang[5]; Ta-Jen Lee[1]; Chi-Neu Tsai[2]; & Cheng-Hsun Chiu[4,6]

----

[1] Division of Rhinology, Department of Otolaryngology, Chang Gung Memorial Hospital and Chang Gung University, Taoyuan, Taiwan

[2] Graduate Institute of Clinical Medical Sciences, College of Medicine, Chang Gung University, Taoyuan, Taiwan

[3] Department of Otolaryngology–Head and Neck Surgery, Chang Gung Memorial Hospital and Chang Gung University, Keelung, Taiwan

[4] Molecular Infectious Disease Research Center, Chang Gung Memorial Hospital, Taoyuan, Taiwan

[5] Department of Thoracic Medicine, Chang Gung Memorial Hospital and Chang Gung Memorial Hospital and Chang Gung University, Taoyuan, Taiwan

[6] Division of Pediatric Infectious Diseases, Department of Pediatrics, Chang Gung Memorial Hospital and Chang Gung University, Taoyuan, Taiwan

METHODS

Immunohistochemistry for IL-17A

Paraffin-embedded adenoid tissue sections were de-waxed in xylene, rinsed in absolute alcohol, and incubated in 3% H_2O_2 for 30 min to quench endogenous peroxidase. The sections were heated in a microwave, incubated in 0.2% normal swine serum (DAKO, CA, USA), and then incubated with specific IL-17A antibody (diluted 1:100) or non-specific purified rabbit IgG (diluted 1:100) for 1 h (LifeSpan BioScience, WA, USA). The avidin-biotin complex method (Vector Laboratories, CA, USA) was used for antibody labeling.

.

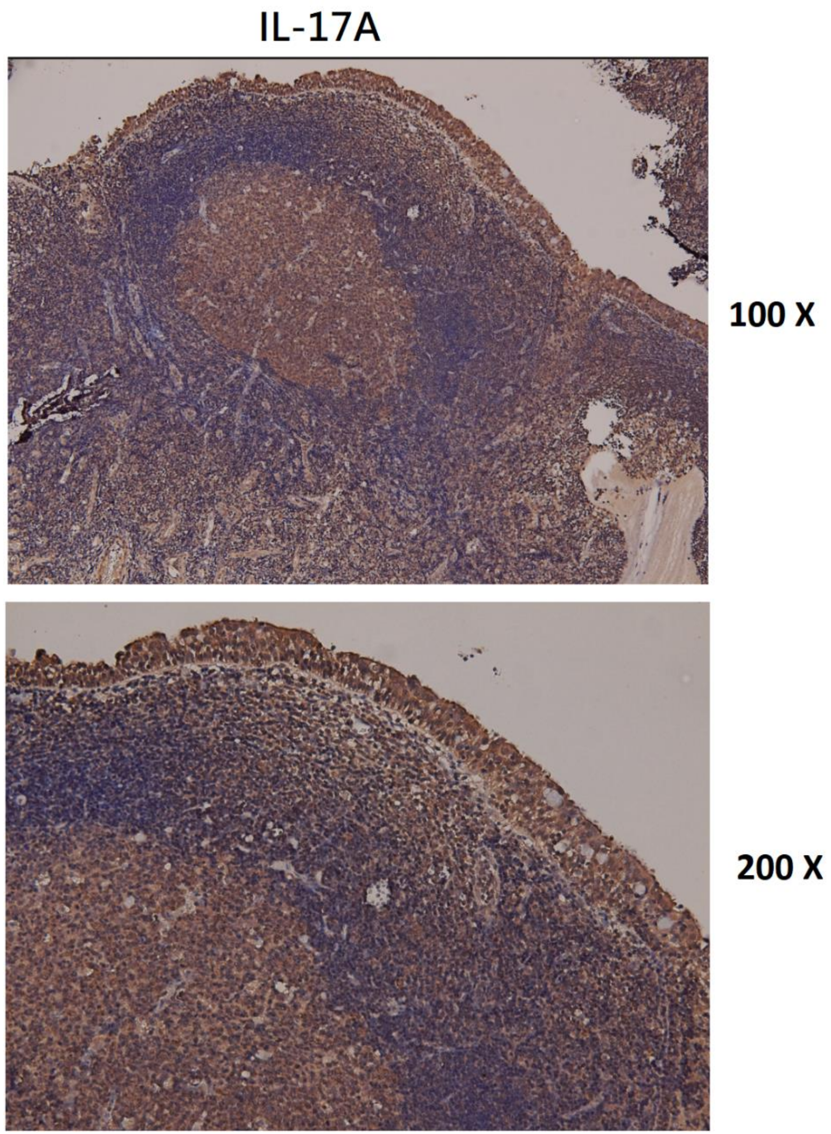

Suppl. Figure 1. Immunohistochemical analysis showing diffuse staining in the epithelium, sub-epithelial tissues, and lymphoid follicles.

	Forward primers	**Reverse primers**
**IL-4**	TTTGCTGCCTCCAAGAACACA	TCCTGTCGAGCCGTTTCAG
**IL-5**	AGACCTTGGCACTGCTTTCT	CAGTACCCCCTTGCACAGTT
**IL-10**	CTCTGATACCTCAACCCCCAT TT	GAGGGAGGTCAGGGAAAACAG
**IL-12A**	TTCACCACTCCCAAAACCTG	AATGGTAAACAGGCCTCCAC
**IL-17A**	TTGGTGTCACTGCTACTGCT	TTGGGCATCCTGGATTTCGT
**RORγt**	CCCAGAACCTCTCTTGGCTT	GAATGGCACAAGTTGGGGTT
**Foxp3**	AGAGAGCCTGCCTCAGTACA	TGACGCTGCTTCTGTGTAGG
**AhR**	TTGTGCCGAGTCCCATATCC	TGGCAGGAAAAGGGTTGGTT
**GADPH**	TTCCAGGAGCGAGATCCCT	CACCCATGACGAACATGGG

Table S1 Primer sequences specific to target genes

RORγt, RAR-related orphan receptor gamma t; Foxp3, Forkhead box P3; AhR, Aryl hydrocarbon receptor**;** GAPDH, glyceraldehyde-3-phosphate dehydrogenase.
